# Arginine Vasopressin-Containing Neurons of the Suprachiasmatic Nucleus Project to CSF

**DOI:** 10.1523/ENEURO.0363-20.2021

**Published:** 2021-04-15

**Authors:** Alana Taub, Yvette Carbajal, Kania Rimu, Rebecca Holt, Yifan Yao, Amanda L. Hernandez, Joseph LeSauter, Rae Silver

**Affiliations:** 1 Department of Psychology, Columbia University, New York, NY 10027; 2 Department of Neuroscience, Barnard College, New York, NY 10027; 3 Department of Pathology and Cell Biology, Graduate Faculty, Columbia University Medical School, New York, NY 10032

**Keywords:** CSF, suprachiasmatic, vasopressin

## Abstract

While it is well established that there are robust circadian rhythms of arginine vasopressin (AVP) in the cerebrospinal fluid (CSF), the route whereby the peptide reaches the CSF is not clear. ***A***, AVP neurons constitute the largest fraction of the SCN neuronal population. Here, we show that processes of AVP-expressing SCN neurons cross the epithelium of the 3rd ventricular wall to reach the CSF (black arrows). Additionally, we report rostro-caudal differences in AVP neuron size and demonstrate that the localization of cells expressing the clock protein PER2 extend beyond the AVP population, thereby indicating that the size of this nucleus is somewhat larger than previously understood. ***B***, Following lateral ventricle (LV) injection of cholera toxin β subunit (CTβ ; magenta) the retrograde tracer is seen in AVP neurons of the SCN, supporting the anatomical evidence that AVP neuronal processes directly contact the CSF.

Arginine vasopressin (AVP) expressing neurons form the major population in the brain’s circadian clock located in the hypothalamic suprachiasmatic nucleus (SCN). They participate in inter-neuronal coupling and provide an output signal for synchronizing daily rhythms. AVP is present at high concentrations in the cerebrospinal fluid (CSF) and fluctuates on a circadian timescale. While it is assumed that rhythms in CSF AVP are of SCN origin, a route of communication between these compartments has not been delineated. Using immunochemistry (ICC) and cell filling techniques, we determine the morphology and location of AVP neurons in mouse and delineate their axonal and dendritic processes. Cholera toxin β subunit (CTβ) tracer injected into the lateral ventricle tests whether AVP neurons communicate with CSF. Most importantly, the results indicate that AVP neurons lie in close proximity to the third ventricle, and their processes cross the ventricular wall into the CSF. We also report that contrary to widely held assumptions, AVP neurons do not fully delineate the SCN borders as PER2 expression extends beyond the AVP region. Also, AVP neurons form a rostral prong originating in the SCN medial-most and ventral-most aspect. AVP is lacking in the mid-dorsal shell but does occur at the base of the SCN just above the optic tract. Finally, neurons of the rostral SCN are smaller than those lying caudally. These findings extend our understanding of AVP signaling potential, demonstrate the heterogeneity of AVP neurons, and highlight limits in using this peptide to delineate the mouse SCN.

## Visual Abstract

**Figure:**
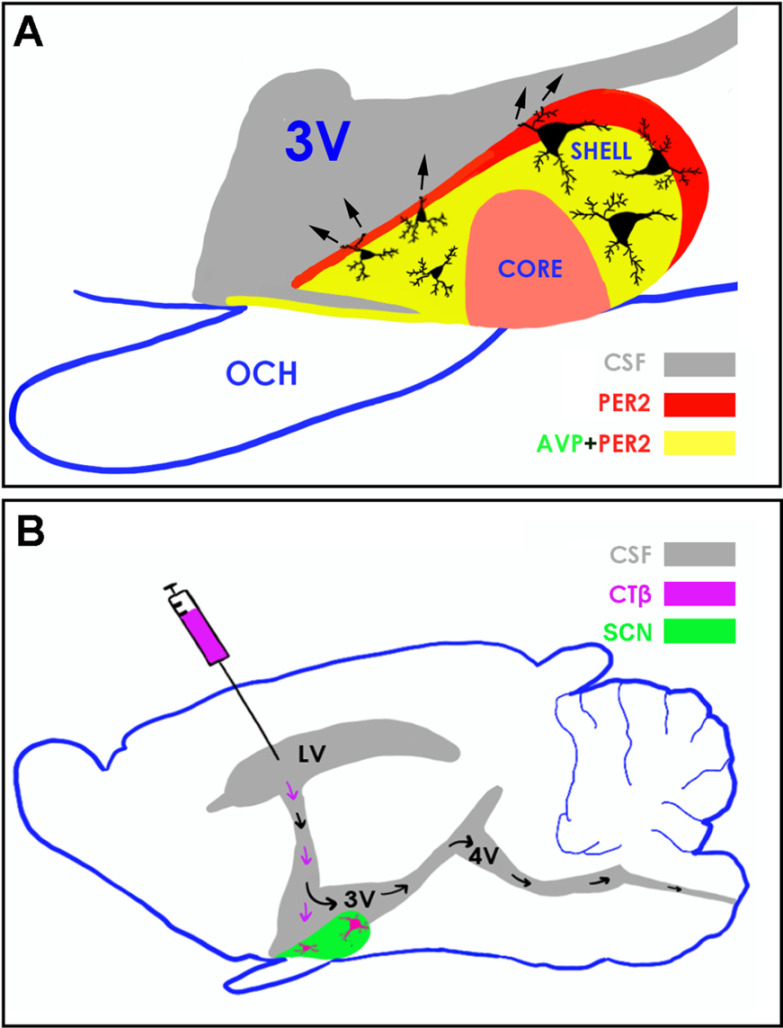

## Significance Statement

There is a high amplitude circadian rhythm of arginine vasopressin (AVP) in the cerebrospinal fluid (CSF), presumed to be of suprachiasmatic nucleus (SCN) origin. There is however, no known route of communication between these compartments. We demonstrate that in the mouse SCN, processes of AVP neurons course toward the third ventricle and cross the ventricular wall to reach the CSF, thereby enabling vasopressinergic signals to reach many brain regions bearing receptors for this peptide. Also, the SCN extends beyond the borders delineated by AVP neurons and rostral AVP neurons are smaller than caudal populations. The work extends our understanding of AVP signaling potential, the heterogeneity of AVP neurons, and highlights limits in using this peptide to delineate the SCN.

## Introduction

The suprachiasmatic nucleus (SCN) of the hypothalamus, comprised of ∼20,000 neurons, functions as a circadian clock, coordinating and synchronizing daily rhythms in numerous bodily functions including metabolism, physiology, behavior, and hormone secretion. These neurons contain a substantial array of peptides (Antle and Silver, 2005), among which the best studied synthesize arginine vasopressin (AVP; van den Pol and Tsujimoto, 1985; Kalsbeek et al., 2010). AVP-expressing neurons form the major population of neurons in the SCN and are estimated to constitute 37% of the nucleus (Moore, 2013). Through most of its rostrocaudal extent, AVP perikarya lie within the shell region (Moore, 2013; Lokshin et al., 2015; Varadarajan et al., 2018). It is widely accepted that AVP neurons delineate the boundaries of the SCN and it is thought that AVP neurons provide the major output signal from the SCN (Cassone et al., 1988; Daikoku et al., 1992; Dai et al., 1997; Abrahamson and Moore, 2001; Ramanathan et al., 2006; Moore, 2013).

### Efferent connections of the SCN

Output from the SCN influences visceral function (Ueyama et al., 1999), REM sleep (Lee et al., 2009), and timing of the LH surge (Williams et al., 2011). SCN efferents have been extensively studied (Moore, 2013; Morin, 2013) and more specifically, the efferents of AVP neurons have also been explored (LeSauter and Silver, 1998; Kalsbeek et al., 2010). The medial preoptic area, the periventricular and sub-paraventricular nucleus, dorsomedial hypothalamus and paraventricular nucleus of the thalamus have been identified as targets of AVP neurons. However, the extent to which all of these connections originate from SCN or AVP neurons of other origins is not known.

### AVP expression in the SCN and CSF is rhythmic

The transcriptional machinery of the core clockwork directly regulates rhythmic expression of AVP in the SCN shell, but not in other AVP-producing neurons (Jin et al., 1999; Silver et al., 1999). The concentration of AVP in the CSF, thought to be of SCN origin, fluctuates daily with a peak in the morning (Schwartz and Reppert, 1985; Söderstein et al., 1985; Jolkkonen et al., 1988; Stark and Daniel, 1989; Kalsbeek et al., 2010). Samples from rat CSF and blood definitively demonstrated circadian rhythms in AVP levels in the CSF but not in the blood (Schwartz et al., 1983). This work established the importance of the SCN in producing circadian rhythms in CSF AVP as SCN lesions abolished rhythmicity and reduced measurable levels of the peptide but left open the possibility that neurons of the SCN may trigger AVP release from some other neural sites. Also unknown was the route whereby AVP of SCN origin might reach the CSF, a question in the present study.

### The present study

While rhythmicity in CSF AVP levels is well established, the question of how AVP of SCN origin might reach the CSF led to the present study. Much of the work on the SCN has been done in coronal sections and this focus neglects the rostral and caudal-most aspects of the nucleus, both of which are rich in AVP containing neurons (Riddle et al., 2017; Varadarajan et al., 2018). Additionally, in coronal sections it is difficult to visualize the close and extended contact of the SCN with the wall of the third ventricle. Here, we first sought to better understand the full extent of AVP neurons in the mouse SCN. This work pointed to dense AVP fibers projecting toward the CSF, a previously unidentified target. To better characterize their axonal and dendritic processes, we filled AVP neurons with biocytin and characterized their afferent and efferent processes using confocal microscopy. To assess communication of AVP neurons with CSF, immunochemistry was used to label the ventricular wall and the tracer cholera toxin β subunit (CTβ), was injected into the lateral ventricle and SCN AVP cells bearing the tracer were identified.

## Materials and Methods

### Animals and housing

Two strains of mice were used to optimize detection of markers of interest with our multi-label protocols. For detection of PER2, we used adult C57BL/NJ mice (The Jackson Laboratory), for cell filling with biocytin, laboratory bred 15-d-old *Per1-*GFP mice (Kuhlman et al., 2000) were used. Mice were group-housed (four per cage) in translucent propylene cages (48 × 27 × 20 cm) in a colony room with a 12/12 h light/dark cycle at 21 ± 2°C and provided with free access to food and water. All procedures were approved by the Columbia University Institutional Animal Care and Use Committee.

### Localization of AVP neurons and efferents

Mice were anesthetized with 100 mg/kg of ketamine and 10 mg/kg of xylazine and perfused transcardially with 50 ml of saline followed by 75 ml of 4% paraformaldehyde. Brains were postfixed overnight at 4°C and then cryoprotected in 20% sucrose in 0.1 m phosphate buffer 0.9% saline (PBS). For immunochemistry (ICC), sections (50 μm) were cut in the sagittal, horizontal or coronal planes on a cryostat (Microm HM 500 M) and collected into PBS. The brain sections were processed as free-floating sections in 24-well plates. For single label ICC, sections were washed in PBS + 0.1% Triton X-100 (PBST) and then blocked with normal donkey serum for 1 h on a shaker at room temperature (RT). Sections were placed in a 0.3% PBST solution containing rabbit anti-AVP antibody at 4°C for 48 h. Sections were washed (3×) and incubated for 2 h at RT in 0.3% PBST containing the appropriate secondary antibody. Sections were washed in PB, mounted, dehydrated in ethanol solutions, cleared in CitriSolve (Fisher Scientific) and coverslipped with Krystalon (EM Diagnostics). For double label ICC, the same procedure was used except primary antibodies were either mouse anti-AVP and rabbit anti-PER2; mouse anti-AVP and rabbit anti-S100β or rabbit anti-AVP and goat anti-CTβ. The appropriate fluorescent secondaries were used (Table 1).

**Table 1:** Primary and secondary antibodies used

Antibodies	Host	Dilution	Company	RRID
AVP	Mouse	1:500	Santa Cruz Biotechnology	sc-390723*
AVP	Rabbit	1:5000	Immunostar	AB_572219
AVP	Guinea pig	1:5000	Peninsula Laboratories	AB_2313978
PER2	Rabbit	1:500	EMD Millipore	AB_1587380
S100β	Rabbit	1:1	Dako	AB_1587380
CTβ	Goat	1:500	Lists Biologicalalals	AB_10013220
Cy2	Rabbit	1:200	Jackson ImmunoResearch	AB_2340612
Cy3	Mouse	1:200	Jackson ImmunoResearch	AB_2340813
Cy3	Rabbit	1:200	Jackson ImmunoResearch	AB_2307443
Cy3	Goat	1:200	Jackson ImmunoResearch	AB_2307351
CY5	Guinea pig	1:200	Jackson ImmunoResearch	AB_2340462
Other products				
Normal donkey serum		1:100	Jackson ImmunoResearch	AB_2337258
NIS Elements software			Morrell Instruments	SCR_002776
Photoshop			Adobe Photoshop CS	SCR_014199

*catalog number as RRID not available.

### AVP in relation to the ependymal wall

To assess AVP fibers in supra- and sub-ependymal zones of the third ventricle, we observed AVP and S100β labeled sections (*n* = 5 female mice). S100β stains ependymal cells (Didier et al., 1986; Carlén et al., 2009; Lavado and Oliver, 2011).

### Biocytin filling of patched AVP cells to determine morphology of AVP neurons

To determine the morphology of AVP neurons and their axonal and dendritic projections, *Per1*-GFP mice were sacrificed by cervical dislocation between zeitgeber time (ZT)12 and ZT15 (when PER1-GFP expression is high), and cells were patched and filled with biocytin as described for GRP-GFP cells (Drouyer et al., 2010). For ICC of sections bearing biocytin-filled cells tissue was incubated overnight at 4°C with Cy2 streptavidin (Jackson ImmunoResearch) in PBST, then processed as above except primary antibodies were guinea pig anti-AVP and rabbit anti-GFP, stained with the appropriate secondary antibodies.

### Analysis of biocytin filling

Each filled cell was examined throughout the z stack using confocal microscopy to assess whether it was single, double or triple labeled for biocytin (green), PER1-GFP (magenta), and AVP (blue). Of 73 cells filled with biocytin in nine animals, 53 were PER1-GFP positive and 20 were PER1-GFP negative. Of the 53 PER 1-GFP positive cells, 30 were AVP+ and 23 were AVP–. The 20 PER1-GFP negative cells were also AVP negative. Axons originate from the soma of AVP neurons or from a proximal dendrite and are very fine and beady. Dendrites are larger and possess spines, swellings, and/or appendages (described in van den Pol, 1980).

### Measuring cell size

In the initial study, confocal microscopy was used to measure the perimeter of distinct populations of AVP neurons, enabling comparison with previous studies of this population (Campos et al., 2014; Moore et al., 2002). Images were taken from the rostral-most and caudal-most aspects, at the largest extent of the SCN, the mid-region in sagittal sections (two sections/SCN, *n* = 8 SCNs). The cells were measured at their greatest extent, where a distinct nucleus was seen on the 1 μm *z*-axis optical section. The perimeter was determined using the Annotations and Measurements tool on NIS-Elements Advance Research software. Seven to 21 cells were measured/region for each brain section (total *N* = 76 rostral, 89 caudal cells). For volumetric measures, a new set of confocal images of AVP stained SCNs were imported into the imaging software Aivia (version 9, DRVISION Technologies LLC). The segmentation output in the pixel classifier tool allows for visualization of the cells in 3D and was used to measure volume of individual rostral (*N* = 88) and caudal (*N* = 101) cells.

### CTβ tracer, lateral ventricle injection site

**Figure 1.**
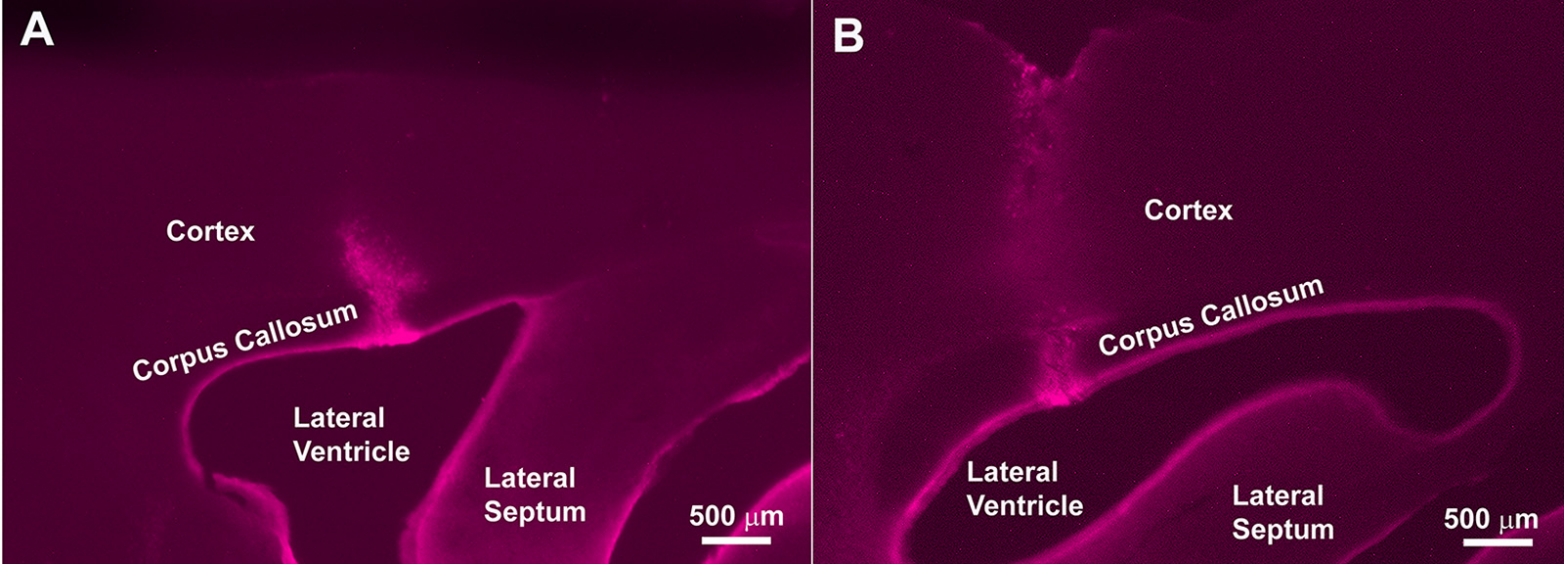
Lateral ventricle injection sites. Light-microscope images of a 50 μm section showing the injection site for CTβ in the lateral ventricle. Some CTβ tracer “en passage” is seen in corpus callosum and cortex, but the SCN does not project to these areas. Injection site in ***A*** is for the SCN shown in Figure 9, and in ***B*** for Figure 10.

While CTβ is an anterograde and retrograde tracer, it behaves primarily as a retrograde tracer at the doses and sites described here (see Leak and Moore, 2012 for extended discussion and explanation). CalB-GFP mice were anesthetized with 100 mg/kg of ketamine and 10 mg/kg of xylazine at ZT14 and prepared for aseptic surgery. The head was shaved and the mouse was positioned in a stereotaxic apparatus (David Kopf Instruments). Injections were aimed at the lateral ventricle (AP +1.1, ML +0.7 from bregma, DV −3.0 from the top of the skull). Pressure injections of 4 μl of CTβ (1% low salt solution, List Biologicals) were made with a 10-μl syringe (Hamilton Co) at a rate of 2 μl/min. The cannula was left in the brain at the end of the infusion for an additional 2 min and removed slowly over 1 min to minimize tracer diffusion upon needle withdrawal. The animals were killed 7 d following surgery. Mice (*n* = 8) were injected with CTβ. The injection penetrated the lateral septum in three brains but was strictly localized to the lateral ventricle in five injections (Fig. 1).

### Microscopy

Immunofluorescent labeling was examined on a Nikon Eclipse E800 (Morrell Instruments) microscope using the filters 480 ± 40 nm for Cy2, 560 ± 40 nm for Cy3 and 620 ± 40 nm for Cy5. Images were captured with a USB-3 DMK33UX174 camera (Morrell Instruments) using the Nikon NIS-Elements Basic Research software. To identify AVP dendrites and axons in biocytin-filled cells, and to determine their relation to the CSF, images were taken on a Nikon Eclipse Ti2E confocal microscope (Nikon) equipped with a LUNV Laser unit and an argon-krypton laser using the excitation wavelengths 488 nm for Cy2, 561 nm for Cy3, and 638 nm for Cy5. NIS-Elements Advanced Research software was used to visualize the z-stacks. Confocal images of sections from CTβ-injected mice were analyzed to determine the colocalization of CTβ with AVP. The images were imported into Photoshop (Adobe Photoshop CS). Single labeled CTβ and AVP and double labeled cells were counted by two experimenters (agreement: 93 ± 2%).

### Image analysis

For visualization of cells and/or fibers, we used Photoshop. To recover lost details in dark and bright areas of the image Shadows and Highlights were adjusted in the Image-Adjustment-Levels dialogue box in Photoshop, by dragging the black and white Input Levels to the edge of the first group of pixels on either end of the histogram (Figs. 4, 5). The function of Shadows and Highlights is to brighten areas of shadow and darken areas of brightness based on surrounding pixels; it does not simply lighten or darken an image (Adobe Photoshop CS6). For color images, Shadows and Highlights were manipulated for each color separately. Grayscale images were inverted before these visual optimizations.

### Statistical analyses

Data for neuronal perimeter and volume of AVP neurons were measured using both statistical inference and estimation statistics. For statistical inference, we used Student’s *t* test for normally distributed data with equal variance, Welch’s *t* test for normally distributed data with unequal variance and Mann–Whitney *U* test for data lacking normal distribution. For these analyses, outliers were removed (for perimeter, 6 rostral and 4 caudal; for volume, 7 rostral and 2 caudal) using the Tukey method [quartile 1 and 3 ± (1.5*interquartile range)]. For both perimeter and volume distribution normality was analyzed by the Kolmogorov–Smirnov test and equality of variances was calculated by *F* test. For perimeter measures, data for both rostral and caudal are normally distributed (D_rostral_ = 0.94, *p* = 0.53; D_caudal_ = 0.09, *p* = 0.46) but have unequal variance (S_rostral_ = 15.21; S_caudal_ = 35.22, *F* = 0.43, *p* = 0.004). Data grouped within individual SCNs are normally distributed (D_rostral_ = 0.22, *p* = 0.75; D_caudal_ = 0.13, *p* = 0.99) and have equal variance (S_rostral_ = 7.8; S_caudal_ = 5.9, *F* = 1.31, *p* = 0.73). For volumetric measures, data for all neurons are normally distributed for rostral cells (D_rostral_ = 0.09, *p* = 0.49), but not for caudal cells (D_caudal_ = 0.14, *p* = 0.02). The volumetric measures within individual SCN are normally distributed (D_rostral_ = 0.17, *p* = 0.98; D_caudal_ = 0.22, *p* = 0.86) and have equal variance (S_rostral_ = 3432.68; S_caudal_ = 11,576.27, *F* = 0.3, *p* = 0.21)]. Estimation statistics were run using https://www.estimationstats.com/. Mean difference was selected for effect size and 5000 bootstrap samples were run using bias‐corrected and accelerated confidence interval (CI) protocols. Date is presented as [95%CI lower, upper range] (Ho et al., 2004).

## Results

### AVP in SCN

**Figure 2.**
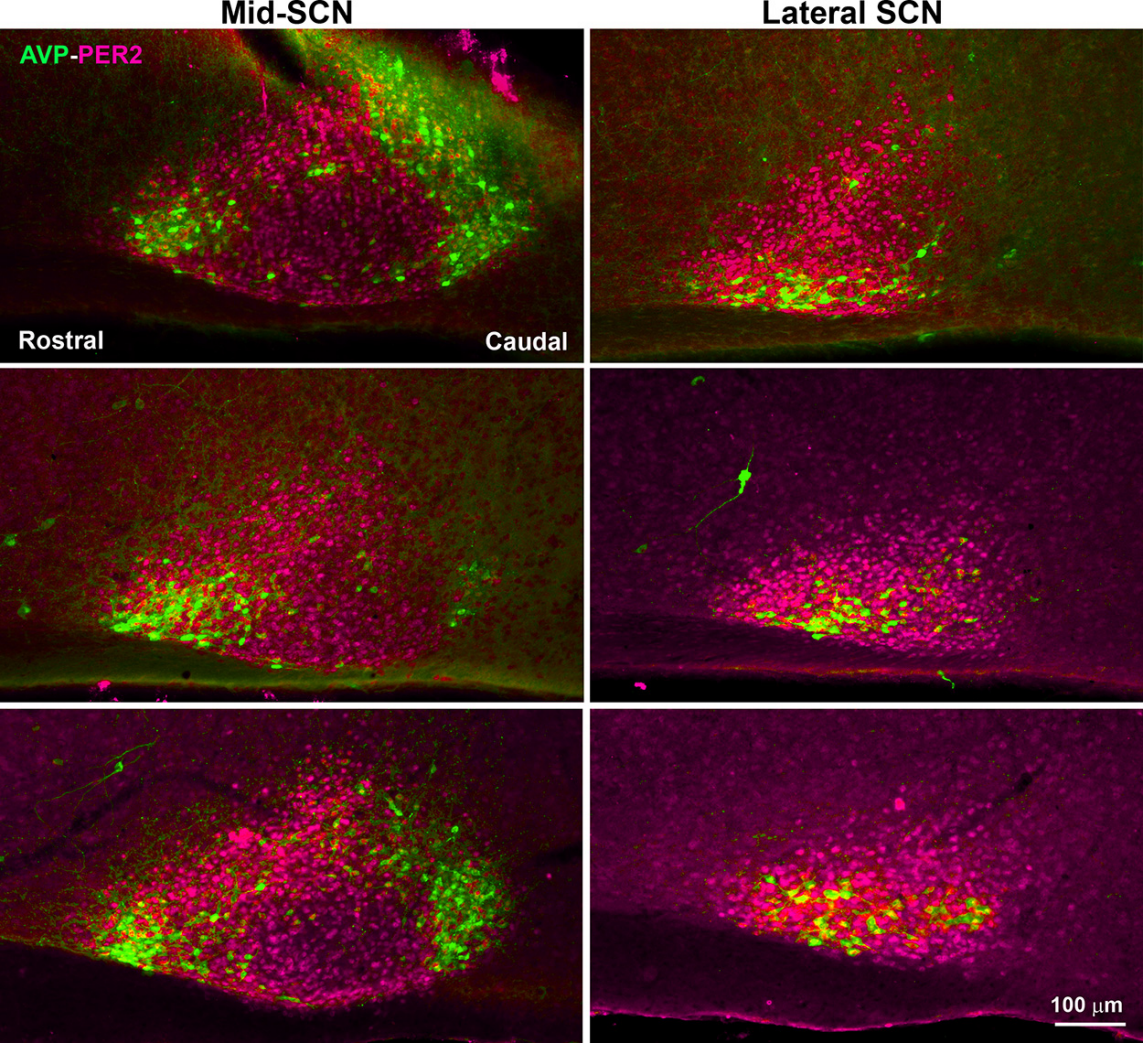
Photomicrographs of sagittal sections at mid (left) and lateral (right) SCN from three representative mice (the most medial aspect of the SCN is not shown). The results indicate that in mid-SCN and lateral-SCN, PER2-positive neurons extend well beyond the borders of AVP neurons. The mice were euthanized at ZT14 (top two panels) and ZT12 (bottom panel).

Because AVP-containing neurons are often used to delineate the extent of the SCN, we investigated the accuracy of this protocol by comparing the area of PER2 expression at its peak (ZT12–ZT14) to that of AVP-ir. In sagittal sections, we were surprised to find that at mid and lateral levels of the nucleus, the area of PER2 expression extends well beyond that delineated by AVP (Fig. 2). Most medially PER2 expression does correspond to the area delineated by AVP neurons (data not shown).

**Figure 3.**
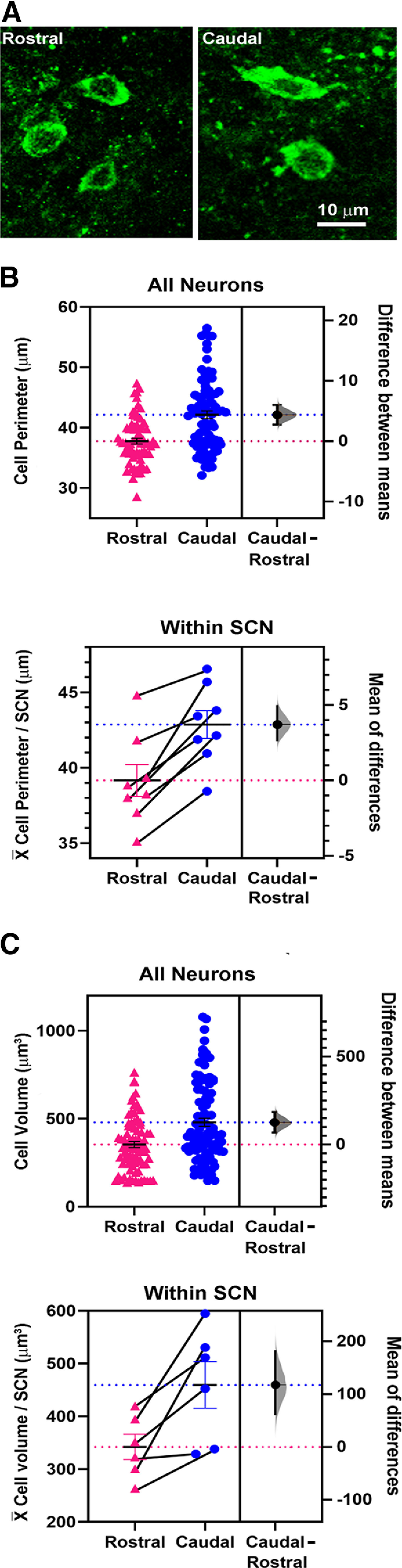
Rostral-caudal differences in AVP neurons. ***A***, Confocal images of representative rostral and caudal cells. ***B***, Top panel, Perimeter ($\bar{X}$± SE) of rostral versus caudal cells in all SCNs. Bottom panel, Perimeter ($\bar{X}$± SE) of neurons within individual SCNs. The lines connect data for rostral and caudal averages within each SCN. ***C***, Top panel, Volume ($\bar{X}$± SE) of rostral versus caudal cells in all SCNs. Bottom panel, Volume ($\bar{X}$± SE) of neurons within individual SCNs. The lines connect data of average volume for rostral and caudal neurons within each SCN. In all panels, for the estimation statistics, the gray curve indicates the entire range of expected sampling error in measuring the mean. The horizontal blue and magenta lines are the means for the caudal and rostral cells respectively. For all measures, the mean for rostral cells lies far outside the curve indicating a significant difference in size compared with the caudal populations.

As previous studies suggest a relationship between the size of SCN neurons and their location within the nucleus (Campos et al., 2014), we also examined the size of AVP neurons in the rostral versus the caudal SCN measuring both perimeter and volume. The results indicate that neurons lying caudally are larger than rostral ones for both measures and this held true whether statistical inference or estimation statistics were applied (Fig. 3).

Measures for perimeter are shown in Figure 3*B*. The statistical results are as follows: for cell perimeter (Fig. 3*B*, upper panel), Welch’s *t*_(69,84)_ = 5.48, *p* = 1.7e-7 [with outliers included, the difference is also significant (Welch’s *t*_(75,88)_ = 3.53, *p* = 0.0005)]. CI unpaired mean difference is 4.36 [95.0%CI 2.84, 5.91], *p* <0.00001. For mean cell perimeter within individual SCNs (Fig. 3*B*, lower panel) Student’s paired *t*_(7)_ = 5.76, *p = 0.0007. Paired mean difference between rostral and caudal is 3.7 [95.0%CI 2.62, 4.94], *p* < 0.00001. For volume per cell (Fig. 3*C*, upper panel), *z*_(81,89)_ = 3.65, *p* = 0.0003 [with outliers included, the difference is also significant (*z*_(87,100)_ = 2.73, *p* = 0.006)]. CI unpaired mean difference = 1.26 × 10^2^ [95.0%CI 72.5, 1.84 × 10^2^], *p* < 0.00001. For mean volume within individual SCNs (Fig. 3*C*, lower panel), Student’s paired *t*_(5)_ = 3.44, *p = 0.02. Paired mean difference = 1.17 × 10^2^ [95.0%CI 61.2, 1.8 × 10^2^], *p* = 0.03.

**Figure 4.**
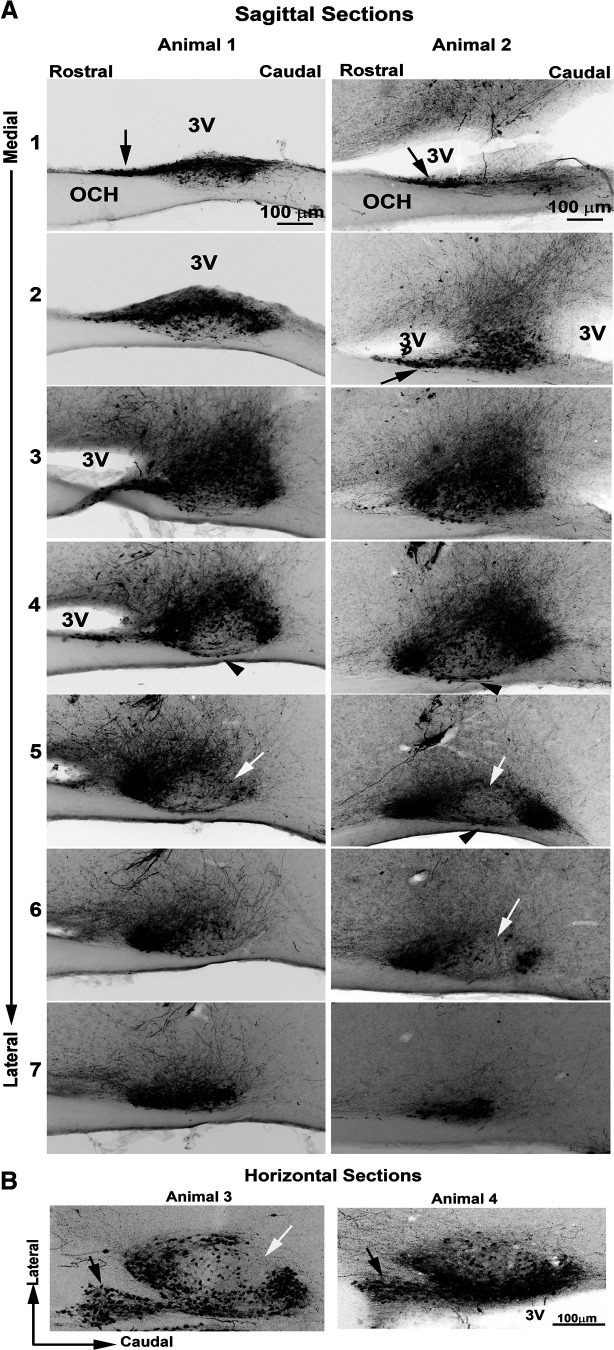
Photomicrographs show the relationship of AVP neurons to the CSF in serial sagittal sections from medial to lateral (***A***) and in horizontal sections (***B***). 3V, third ventricle; OCH, optic chiasm.

The precise localization of AVP neurons in the main body of the SCN is shown in seven serial sagittal sections (50 μm) through the extent of the SCN from the medial-most to the lateral-most aspect, in two animals sectioned at slightly different angles. Additionally, a substantial rostral prong is shown in both sagittal and horizontal views (Fig. 4).

While the SCN is embedded within the hypothalamus for most of its extent, most medially, it is separated from the rest of the hypothalamus by the third ventricle (Fig. 4, animals #1 and #2, images 1 and 2). At its dorsal aspect the SCN abuts the third ventricle, most evident when the section is cut perfectly parallel to the midline (Fig. 4, animal #1, images 1 and 2). Here the medial SCN is located right below the third ventricle. At the mid-level (animal #1, images 3–5), the rostral pole of the SCN abuts the third ventricle and the main body is caudal to the ventricle. Laterally (animal #1, image 7), the SCN lies entirely within the hypothalamic parenchyma and is no longer in contact with the third ventricle. In animal 2, in sections cut at a different angle, the third ventricle is seen medially (images 1–3), but no longer visible at images 4–7.

At the medial aspect of the SCN, AVP neurons occur throughout the SCN (Fig. 4*A*, animal #1, image 1; animal #2, images 1 and 2), and these extend in a rostral prong that originates in the ventral part of the nucleus and lies below the third ventricle, just above the optic chiasm (sagittal view in Fig. 4*A*, animal #1, image 1, animal #2, images 1 and 2; and horizontal views in Fig. 4*B*, animals #3 and #4, black arrows). The rostral prong extends ∼250 μm rostrocaudally, 20–30 μm dorsoventrally, and ∼80 μm mediolaterally. At the mid-SCN at its greatest extent in the sagittal view, AVP neurons lie rostrally and caudally, but are sparse in the core and in the mid-dorsal aspect (animal #1, image 5; animal #2, images 5,6; see also horizontal view animal #3 {white arrows}). In addition, AVP neurons are present along the base of the SCN (Fig. 4, both animals, image 4; animal #2, image 5, arrowheads). At the lateral aspect, the AVP neurons are densely packed (both animals, image 7).

### Relation of AVP fibers to ependymal wall and CSF in coronal and sagittal sections

**Figure 5.**
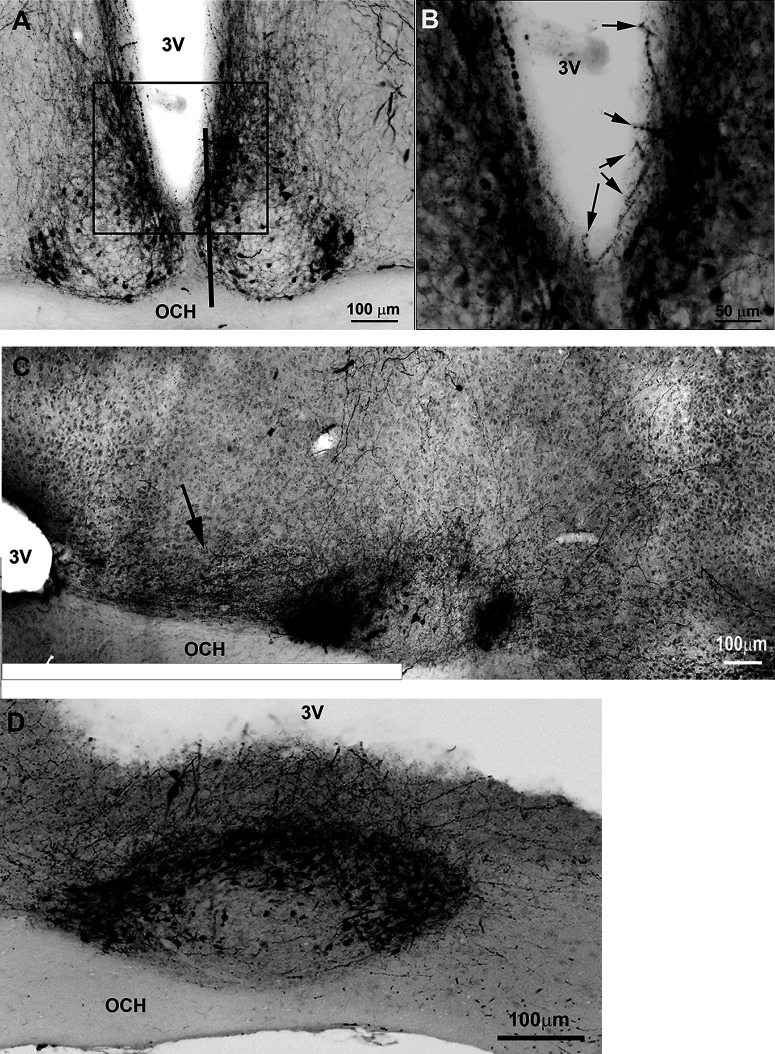
Photomicrographs of coronal (***A***, ***B***) and sagittal (***C***, ***D***) sections through the SCN show extensive AVP projections coursing toward the CSF. ***A–C***, Light-microscopic images of 50 μm sections and ***D*** is a confocal image (*z*-axis: 2 μm) ***A***, Image of a coronal section of the SCN, stained for AVP showing fibers lying close to CSF. The black box delineates the location of the image in ***B*** shown at a higher magnification. The black line points to the approximate location of a sagittal section shown in ***D***. ***B***, Higher magnification of the same SCN shown in ***A***. Arrows point to AVP fibers projecting to the third ventricle. ***C***, Sagittal section through mid SCN shows AVP-ir fibers projecting rostrally toward the third ventricle (black arrow). ***D***, Confocal image of a sagittal section at the level of the medial SCN [lies at location shown in Figure 4 (animal #1, image 2)]. Many fibers can be seen coursing toward and possibly crossing the ventricular wall from the SCN to the CSF (*z*-axis: 2 μm). 3V: third ventricle; OCH: optic chiasm.

In coronal sections, at its greatest extent, the SCN is rich in AVP neurons (Fig. 5*A*), and many dendrites and axons course toward the CSF (Fig. 5*B*). In sagittal sections, projections of AVP neurons travel rostrally toward the third ventricle (Fig. 5*C*, arrow). Close to the midline, the SCN lies directly above the optic chiasm and its dorsal aspect abuts the third ventricle (Fig. 5*D*). Here, AVP fibers, appear to cross the ependymal wall to reach the CSF in the third ventricle.

### Morphology of axons and dendrites of AVP neurons

**Figure 6.**
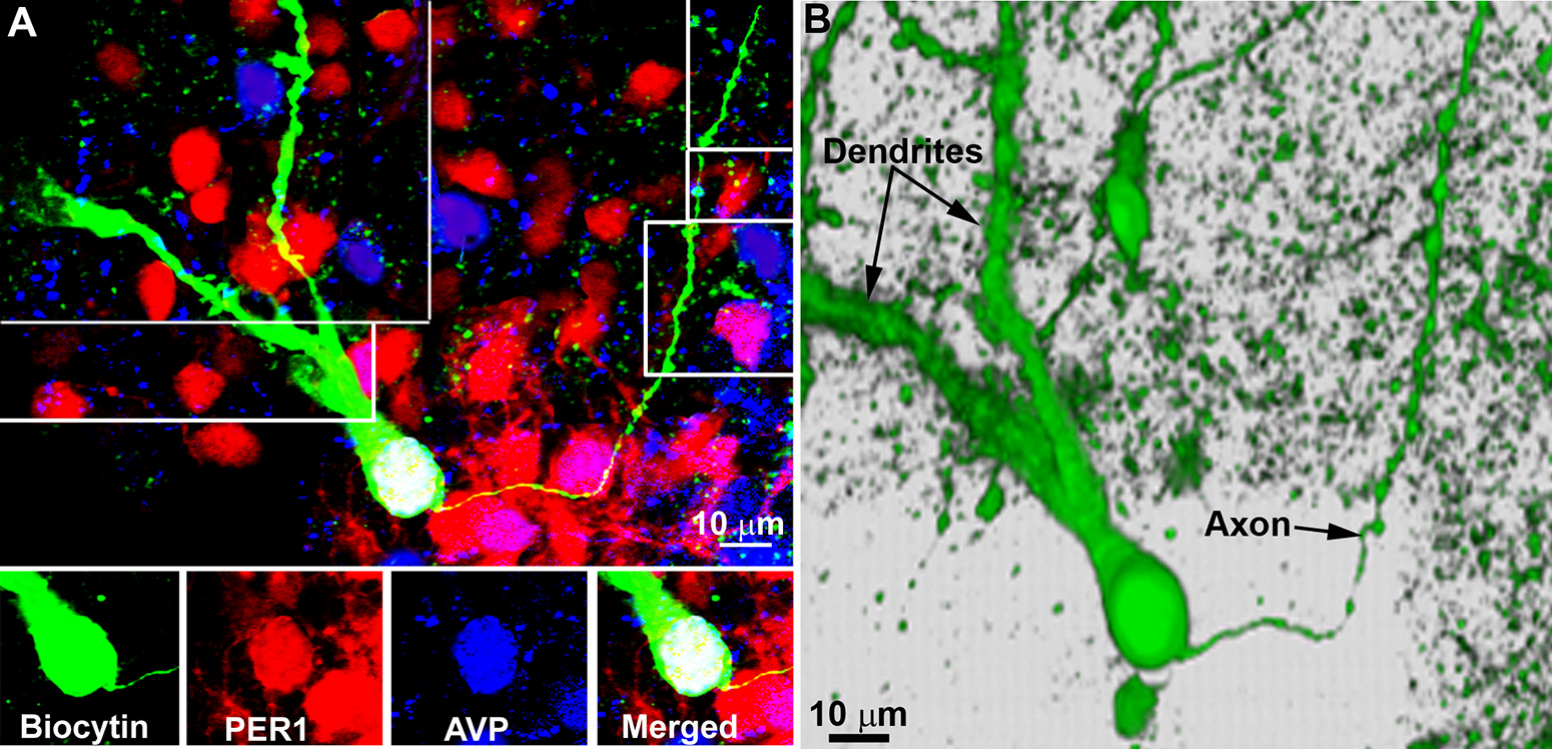
***A***, Peptidergic content of a biocytin-filled AVP neuron. The montage is created by aligning 1 μm *z*-axis confocal images over a *z*-axis of 18 μm as the process courses through the tissue. The bottom row shows the individual peptides within the biocytin-filled cell. ***B***, 3D image of the same cell highlighting the distinct morphologic features of axons and dendrites. The axon is thin and beady and the dendrites are thicker and bear varicosities and spines.

To identify the axons and dendrites of AVP neurons, we patched and filled SCN neurons with biocytin. Of the 30 AVP+ neurons studied, 22 had distinguishable axons and dendrites. As previously described (van den Pol, 1980; Abrahamson and Moore, 2001), axonal processes of SCN origin are very thin and exhibit bulbous, irregularly spaced varicosities along their entire length (Fig. 6). Dendrites are larger and possess spines, swellings and/or appendages.

### SCN dendrites and/or axons crossing the ependymal wall

**Figure 7.**
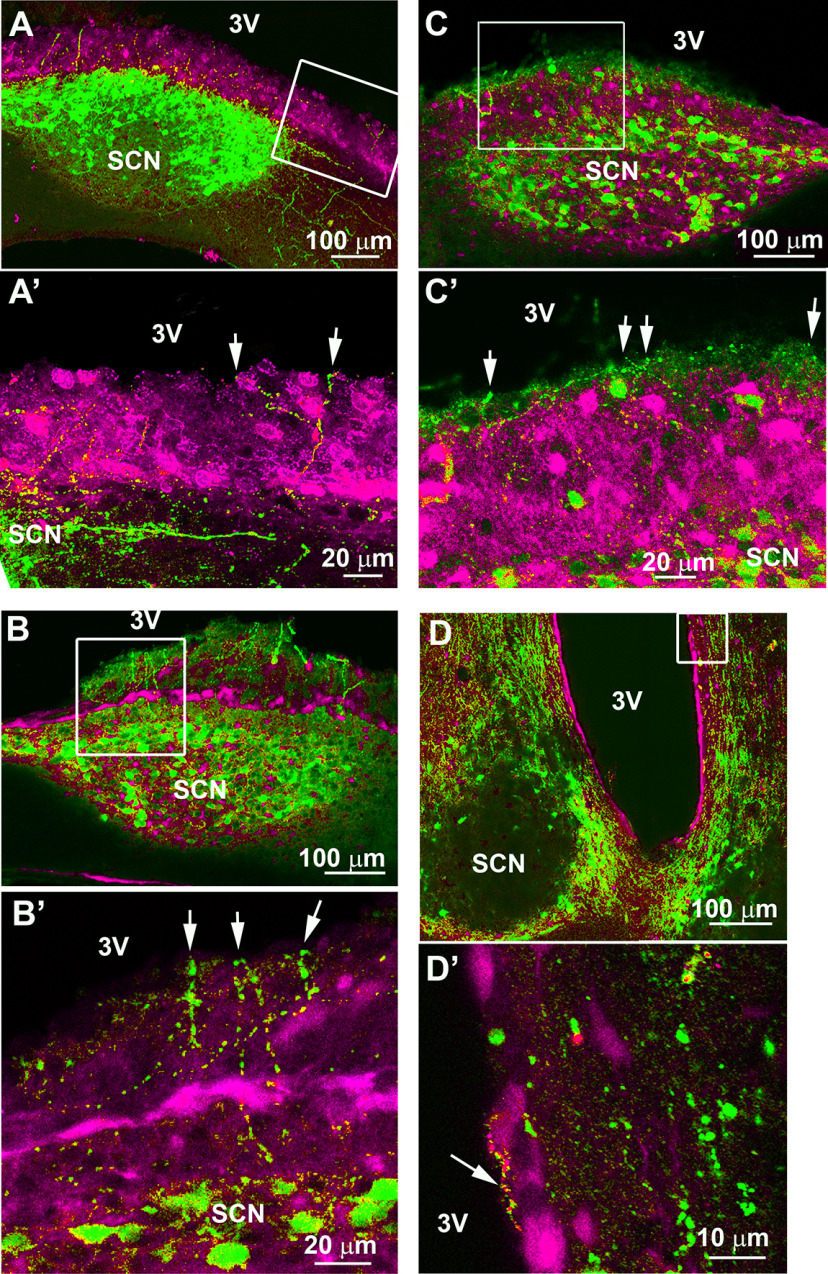
Confocal images of sagittal (***A*–*C***) and coronal (***D***) sections stained for AVP (green) and S100β (magenta), a marker of the ependymal wall. Axons at the level of the SCN cross the ventricular wall into the third ventricle. Boxes in ***A–D*** show the areas enlarged in ***A’–D’***. ***A***, Section of the medial SCN (level 2 in Fig. 4) showing many fibers crossing the ventricular wall (*z*-axis: 2.6 μm). ***A’***, Image showing the two branches of an axon reaching the CSF, caudal to the SCN (arrows; *z*-axis: 1 μm). ***B***, ***C***, Sections (at level 2 in Fig. 4) show AVP fibers extending dorsally from the SCN, crossing the ependymal wall (*z*-axis: ***B***, 2.6 μm; ***C***, 3.5 μm). ***B’***, ***C’***, Images showing axons crossing the ependymal wall with terminal boutons ending in the third ventricle (arrows; *z*-axis, ***B’***, ***C’***, 1.6 μm). ***D***, Section showing the SCN and the third ventricle (*z*-axis: 1 μm). ***D’***, High-magnification images of the area dorsal to the SCN showing an AVP axon coursing on the ventricular side of the ependymal wall (*z*-axis: 0.5 μm). SCN: suprachiasmatic nucleus; 3V: third ventricle.

**Figure 8.**
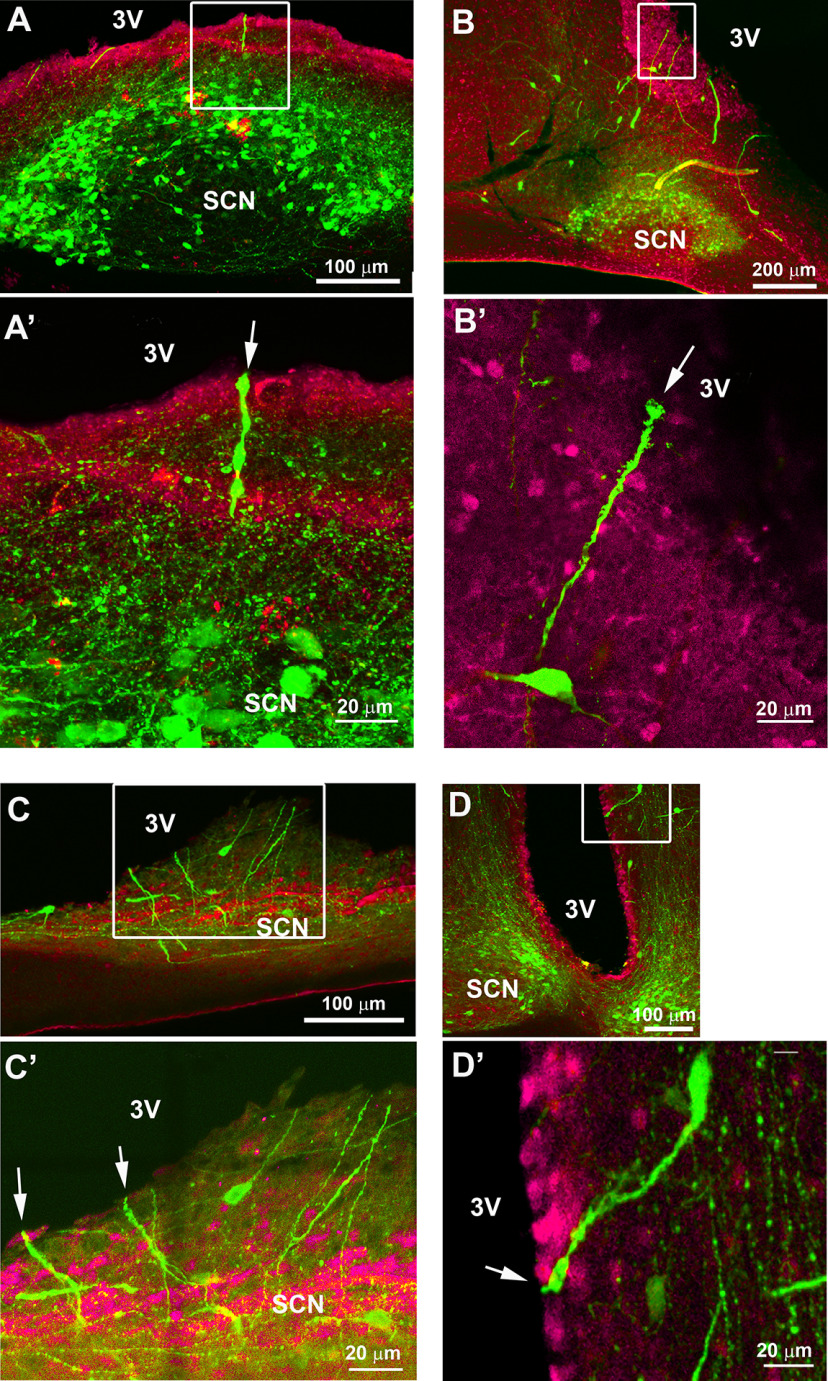
Confocal images of sagittal (***A*–*C***) and coronal (***D***) sections stained for AVP (green) and S100β (magenta), a marker of the ependymal wall showing dendrites at the level of the SCN and nearby, crossing the ventricular wall into the third ventricle. Boxes in ***A–D*** show the areas enlarged in ***A’–D’***. ***A***, Section of a mid SCN (image 2 in Fig. 4) showing fibers crossing the ventricular wall (*z*-axis: 7 μm). ***A’***, Image showing a large AVP dendrite terminating in the CSF by a bulbous swelling (arrow; *z*-axis: 4 μm). ***B***, AVP dendrites extending toward the third ventricle (*z*-axis: 50 μm). ***B’***, Image showing a dendrite terminating in the third ventricle by a large terminal bulb (arrow; *z*-axis: 0.25 μm). ***C***, Sagittal section of a medial SCN (image 1 on Fig. 4) showing fibers extending toward the third ventricle (*z*-axis: 16 μm). ***C’***, Image showing two dendrites terminating in the third ventricle (arrows, *z*-axis: 2.5 μm). ***D***, Coronal section showing the SCN and the third ventricle (*z*-axis: 4.3 μm). ***D’***, Image showing a large dendrite, dorsal to the SCN, crossing the ventricular wall, ending in the third ventricle (arrow, *z*-axis: 2.5 μm). SCN: suprachiasmatic nucleus; 3V: third ventricle.

To determine the terminal regions of SCN AVP neurons, we labeled sagittal sections for AVP and S100β, an ependymal wall marker. Some processes appear to terminate subependymaly while others cross into the third ventricle. Both AVP axons (Fig. 7) and dendrites (Fig. 8) extend through the ventricular wall both at the level of the SCN (Figs. 7*A–C*, 8*A*,*C*) or near the SCN (Figs. 7*D*, 8*B*,*D*). Terminal boutons can be seen within the third ventricle (Fig. 7*B’*,*C’*).

### CTβ tracer in lateral ventricle labels SCN neurons

**Figure 9.**
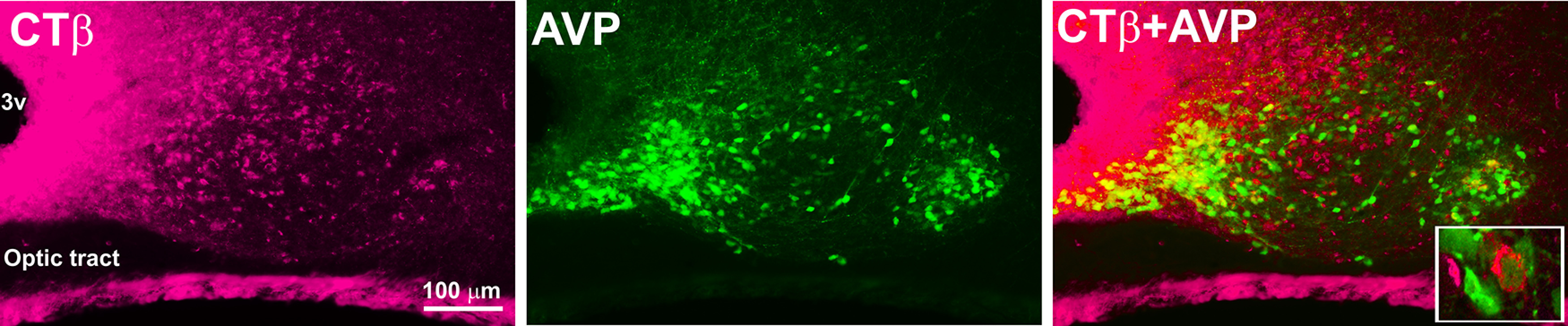
Confocal images (*z*-axis: 4 μm) of an SCN stained for CTβ (magenta), AVP (green), and double-labeled. Inset, Image of a cell double labeled for CTβ+AVP (*z*-axis: 1 μm).

As noted by Leak and Moore (2012): “The CSF contains neuroactive substances that affect brain function and range in size from small molecule transmitters to peptides and large proteins. CSF-contacting neurons are a well-known and universal feature of non-mammalian vertebrates…” Given the evidence of AVP+ fibers directed to the CSF, we next examined whether AVP cells would uptake the tracer CTβ from the ventricle. For this, we injected CTβ into the lateral ventricle. Figure 9 shows the cells that took up the CTβ tracer and colocalized with AVP (inset) in the mid SCN. The distribution pattern of CTβ in the SCN was similar in all five animals where injection was restricted to the lateral ventricle.

Of the 4122 AVP and 2488 CTβ cells evaluated, 15.3 ± 0.9% AVP were co-labeled with CTβ and 25.5 ± 1.9% CTβ cells were co-labeled with AVP. The results point to extensive input from CSF to SCN neurons bearing AVP.

### CTβ in SCN medial to lateral

**Figure 10.**
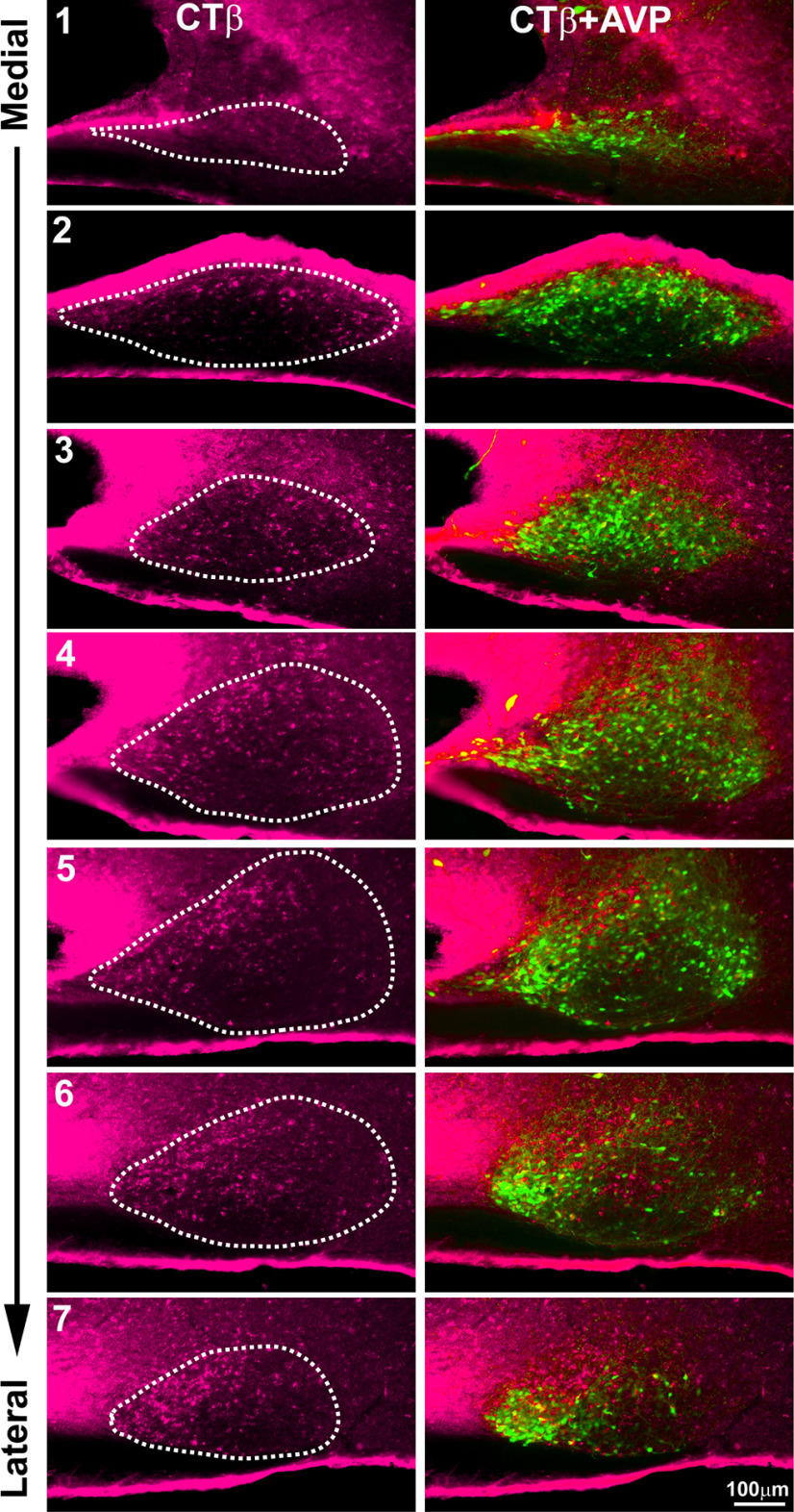
The photomicrographs show the full extent of the SCN in sagittal view from its medial to lateral-most aspect in sections labeled for CTβ (magenta, left column), and CTβ+AVP (right column). The SCN, outlined in white dots, indicates the area used to count cells.

Cells containing CTβ are located mostly within the SCN shell where AVP cells are present (Fig. 10). There are a few CTβ cells in the very medial SCN (image 1). They are located dorsally in the medial SCN (image 2). In the mid and lateral SCN, they are located mostly within the dorsomedial region and are sparse in the SCN core (images 5–7). They are also numerous in the dorsal area where AVP is sparser (image 5).

## Discussion

### AVP fibers reach the CSF

A major finding of the present study is that SCN AVP neurons course toward and even terminate in the fluid space of the third ventricle. This communication is most obvious in sagittal sections through the SCN, especially near the midline. And while AVP neurons have often been used to demarcate the outer borders of the SCN (Vandesande et al., 1975; Sofroniew and Weindl, 1980; Card and Moore, 1984; van den Pol and Tsujimoto, 1985), the sagittal view makes it clear that there are rhythmically expressing PER2 neurons that lie beyond this demarcation, especially at the lateral borders of the nucleus.

Taken together the detailed description of AVP neurons in the SCN make three distinct points: AVP cells are at the base of the core region of the SCN (Fig. 4). Dorsomedial region of the SCN shell is devoid of AVP neurons, AVP neurons extend rostrally along the midline along a narrow protrusion that originates in the medial-most aspect of the ventral SCN. While each of these details is modest, taken together they can correct incomplete or erroneous anatomic information in previous less detailed work and can provide the groundwork for fine-tuning future studies. Among the questions raised is the nature and function of rhythmic SCN neurons that lie around the outer borders of the SCN and whose existence was not previously realized.

As noted in the Introduction, efferent projections of the SCN and more specifically, efferents of AVP neurons of the SCN have been extensively studied (Berk and Finkelstein, 1981; Stephan et al., 1981; Hoorneman and Buijs, 1982; Watts and Swanson, 1987; Watts et al., 1987; Kalsbeek et al., 1993; Morin, 1994; Teclemariam-Mesbah et al., 1999; Leak and Moore, 2001; Moore and Silver, 1998). Although there are some differences among species (Morin, 1994), AVP is a major component of the SCN in most species that have been studied (Morin and Allen, 2006). Furthermore, an important contribution of SCN to rhythmic AVP levels in CSF has been known since the mid-1980s, although evidence of the route of communication was lacking. To our knowledge however, there are no prior reports of SCN processes reaching the CSF, though there is light and electron microscopic evidence of (unidentified) somal processes inserted in the ependymal layer or directly contacting the CSF space (Xiao et al., 2005). This may be a consequence of the difficulty of detecting the very fine SCN fibers that course in a rostro-caudal direction (Figs. 4, 5) especially when they lie right near the midline. The results are consistent with evidence of volume transmission by the SCN and with prior studies. Specifically, paracrine signaling with a role for AVP, has been demonstrated in culture preparations of the SCN [Honma, 2020 (see Fig. 5), Maywood et al., 2011; Tokuda et al., 2018]. Furthermore, SCN grafts encapsulated in a polymer material can restore circadian activity rhythms through volume transmission (Silver et al., 1996). Finally, the SCN is densely labeled with CSF injections of >20 nl of a tract tracer CTβ (Leak and Moore, 2012). More specifically, they showed that within 2–10 d of injection, the CTβ label appears in specific groups of neuronal perikarya in the telencephalon, thalamus, hypothalamus and brainstem, many at a considerable distance from the ventricles. They hypothesize that these observations support the view that ventricular CSF is a significant channel for volume transmission and identify those brain regions most likely to be involved in this process.

### AVP neurotransmitters

The role of AVP in rhythmicity has been puzzling in view of the fact that AVP-deficient Brattleboro rats display attenuated but normal circadian rhythms (Groblewski et al., 1981; Brown and Nunez, 1989; Kalsbeek et al., 2010). AVP-containing neurons express GABA and prokineticin 2 (Masumoto et al., 2006; Welsh et al., 2010) pointing to the possibility that these neurons play a broader role in the circadian timing system than does the AVP protein itself. Mice in which the essential core clock gene *Bmal1* was deleted in AVP neurons (Avp-Bmal1^−/−^ mice), have impaired circadian activity rhythms (Mieda et al., 2015) and a gradually expanded interval activity time on being moved from LD to DD. Bmal1 restoration in AVP neurons of the SCN using a recombinant adeno-associated virus vector reversed the circadian impairment of Avp-Bmal1^−/−^ mice. The results were interpreted to mean that the circadian oscillation persists at the cellular level, but that mutual coupling among SCN neurons that regulate activity onset and offset was impaired.

### Rhythms in SCN-regulated functions

As noted by van den Pol (2012) peptide release is difficult to study, as it is seen independent of synaptic specializations, in dendrites, axonal boutons and axon shafts. This is consistent with the possibility that axons and dendrites described here (Figs. 7, 8) both contribute to the rhythms of AVP in CSF. AVP has a role in the regulation of hydration, blood pressure, body temperature, corticotropin release, and has been implicated in depression, memory, social and sociosexual behavior and in sex differences (for review, see de Vries and Miller, 1998; Kalsbeek et al., 2010). The stress system in the human brain has been implicated in depression and neurodegeneration (Swaab et al., 2005). The relationship of CSF levels of AVP to plasma levels is of interest in studies of autism, suicide (Brunner et al., 2002) and personality disorders (Coccaro et al., 1998; Oztan et al., 2018) and the two compartments seem to be separately regulated (Kagerbauer et al., 2013). Much remains to be investigated as understanding the function of this compartment in the context of circadian rhythms requires unravelling a sequence of steps involved in volume transmission. AVP is a phylogenetically ancient molecule of immense versatility with very diverse functions in both the brain and in the periphery (Zelena and Engelmann, 2018). Within the SCN this peptide is important not only as an output signal but also in maintaining synchrony of the SCN network (Shan et al., 2020). A broad view of its potential was suggested decades ago when Gillette and Reppert (Gillette and Reppert, 1987) hypothesized that “Oscillations in AVP secretion and neuronal firing rate potentially represent separate modes by which the SCN transmit time information to other brain regions. The electrical oscillation may relay time-of-day by efferent neuronal output along a limited number of specialized circuits. The AVP secretory rhythm potentially modulates daily rhythm in physiology by a generalized neurohormonal effect.” The present work directs attention to the latter possibility.

## Synthesis

Reviewing Editor: Douglas Bayliss, University of Virginia School of Medicine Decisions are customarily a result of the Reviewing Editor and the peer reviewers coming together and discussing their recommendations until a consensus is reached. When revisions are invited, a fact-based synthesis statement explaining their decision and outlining what is needed to prepare a revision will be listed below. The following reviewer(s) agreed to reveal their identity: Eric Bittman.

This manuscript was seen by the same two reviewers who again noted the high quality of the anatomical work, which will be of interest to a specialized audience interested in SCN. The revised manuscript addressed many of the initial concerns (e.g., statistics), although it did not take on the more substantive suggestion to provide additional analysis into the functional significance of the anatomical findings. Nonetheless, the value of these purely anatomical studies was recognized. The reviewers provide additional comments that should be addressed in a revised manuscript. Of particular importance, the possibility that this work may pertain only to mouse should be explicitly acknowledged, especially since circadian research is performed in other species (e.g., hamsters).

Please find the full comments appended below.

Reviewer #1

Advances the Field

Descriptive work showing apparently unknown projections from SCN Avp neurons to the 3rd ventricle with standard methods

Statistics

Needs clarification if the data was normally distributed with equal variance, how outliers were defined, and if the conclusion changes when outliers are included

Visual Abstract

The visual abstract summarizes the paper, but I would suggest making the 3V label and arrow more obvious (dark color, thick arrow, and large font for 3V; lighter colors, thin arrows, smaller font for the rest), since that is the new result here.

Comments

Several changes are actually not highlighted in red, but seem to be minor (parts moved from the figure legends into the main text, shortened parts of the previous text, removing a reference).

The new visual abstract is helpful, but I would suggest making the 3V label and arrow more obvious (dark color, thick arrow, and large font for 3V; lighter colors, thin arrows, smaller font for the rest), since that is the new result here.

The GRP data that is now in Extended Fig. 9-1 is not addressed or discussed in the main text - is it still necessary?

The new 3D voxel view of the raw data does not help visualize the morphology of that cell (I was thinking of a threshold-based surface reconstruction of only that cell), so it could be removed again.

The Fig. 6C box error the authors are referring to in the rebuttal is not clear. The box and the picture are the same, only with more green contrast, and can remain as they are.

The authors added the purpose they used the Photoshop functions for, not what these functions do. There are still grammar issues, but since the text is easy to understand, this is not important and should not delay publication.

Reviewer #2

Advances the Field

Provides further anatomical information about a functionally important hypothalamic nucleus that will be of interest to specialists. Does not supply additional advances in the physiology so that the implications of these descriptive studies are suggestive.

Visual Abstract

Visual abstract is appealing and helpful as a review although the specific efferent projections (blue arrows) to multiple preoptic, hypothalamic, and thalamic areas are not documented in this study, and more distant efferent projections are omitted.

Comments

I agree whole-heartedly that purely anatomical studies constitute significant contributions and I find the figures to be of high quality; the work is well-done and of interest to me as an afficionado of the SCN and circadian rhythms. However, in the absence of physiological data to complement this work, I continue to question whether this contribution belongs in a more specialized journal. The addition of volumetric measurements, clarification of image processing, and statistical revisions are appreciated and improve the ms but do not address the fundamental issues raised in the first review. Furthermore, I have some concerns about the direct applicability of these findings to earlier evidence on CSF-mediated (humoral) communication of circadian signals of SCN origin.

There is considerable variation between species in the anatomical distribution of peptidergic cell types of the SCN (Morin JBR 22:3-13, 2007; Exp Neurol 243:4-20, 2013), with the mouse being less clearly compartmentalized than the hamster, in which the evidence from encapsulated transplants provides the most compelling evidence for humoral signaling. Some of the other evidence for this route comes from primates; the mouse evidence is not as strong. The authors don't consider the possibility that the SCN organization they report, with an absence of AVP-ir in the mid-dorsal SCN, as specific to mouse. Surprisingly, it's not until the methods section (l. 111) that the authors identify the mouse as the subject and possibly the only species to which their findings apply.
